# Supplementation with Vitamins C and E and Exercise-Induced Delayed-Onset Muscle Soreness: A Systematic Review

**DOI:** 10.3390/antiox10020279

**Published:** 2021-02-12

**Authors:** María F. Torre, María Martinez-Ferran, Néstor Vallecillo, Sergio L. Jiménez, Carlos Romero-Morales, Helios Pareja-Galeano

**Affiliations:** 1 Faculty of Sport Sciences, University Europea de Madrid, Villaviciosa de Odón, 28670 Madrid, Spain; mtorr272@fiu.edu (M.F.T.); maria.martinez.nutricion@gmail.com (M.M.-F.); carlos.romero@universidadeuropea.es (C.R.-M.); 2 Faculty of Biomedical and Health Sciences, University Europea de Madrid, 28670 Madrid, Spain; nestor.vallecillo@universidadeuropea.es; 3 Centre for Sport Studies, University Rey Juan Carlos, Fuenlabrada, 28943 Madrid, Spain; sergio.jimenez.saiz@urjc.es

**Keywords:** antioxidant, oxidative stress, performance, skeletal muscle

## Abstract

Muscle damage induced by exercise may have several consequences such as delayed-onset muscle soreness, a side-effect of the release of free radicals during oxidative stress. To mitigate the oxidative stress cascade, the oral intake of antioxidants has been assessed by several research groups. This review examines whether supplementation with vitamin C and/or vitamin E is able to prevent or attenuate delayed-onset muscle soreness after eccentric exercise. The PubMed, Web of Science, Medline, and Embase databases were searched to identify studies meeting the inclusion criteria: primary randomized control trials, healthy male and female participants aged 16–80 years, and an intervention consisting of the intake of vitamin C and/or vitamin E without other supplements plus a controlled eccentric exercise regimen. Further requirements were the measurement of muscle soreness or markers of delayed-onset muscle soreness. All original full-text articles in English or translated into English published from January 2000 to June 2020 were considered for this review. Fourteen studies were finally identified, including 280 participants, 230 men, and 50 women aged 16–30 years. All participants were healthy individuals with different starting levels of physical activity. Supplementation was acute in two studies and chronic in 12, and its consisted of vitamin C in eight studies, vitamin E in two studies, and both in four studies. Only in 3 of the 14 studies was muscle soreness found to be significantly reduced in response to vitamin C and/or vitamin E supplementation at all time points when compared to the placebo group. Despite some studies showing the beneficial effects of chronic supplementation with these vitamins on muscle soreness manifesting 24–72 h after eccentric exercise, the evidence is so far insufficient to confirm that the intake of antioxidant vitamins is able to minimize delayed-onset muscle soreness in this context.

## 1. Introduction

Today, more and more people are taking on a sport in an effort to improve their level of fitness. In parallel with this trend, sports nutritionists are becoming more aware of the need for different nutritional techniques to resolve the challenges of metabolic adaptations in sports enthusiasts. Delayed-onset muscle soreness (DOMS) is a cascade phenomenon with negative consequences for both individuals who are physically active and those who have been inactive for long periods and wish to return to professional sports, return to training regimens, or simply improve their level of physical activity [1]. DOMS is the pain and stiffness felt several hours after strenuous or unaccustomed exercise [2]. Muscle soreness commences 12–24 h after exercise, peaks 24–72 h later, and lasts for five-to-seven days [3]. DOMS is considered a grade 1 muscle strain injury and has sparked interest among researchers who have focused on deciphering its etiology and finding a way to resolve this problem. Though there is no single explanation for the pathophysiological mechanism underlying DOMS, one theory evokes ultrastructural damage to muscle fibers, which leads to the apoptosis and further degradation of these protein cells [4]. Such structural damage of the sarcomere could be related to the recruitment of inflammatory response cells such as neutrophils, macrophages, and cytokines triggered by a higher concentration of calcium in skeletal muscle ion channels. This exerts a greater demand on connective tissue, along with an increased stimulation of never endings and pressure by mechanoreceptors [5]. This cascade could be responsible for the severe muscle tenderness and soreness produced by DOMS. Other studies have shown that DOMS may be linked to other muscular responses to exercise such as inflammation, injury, high temperatures, spasms, lactic acid levels, and connective tissue damage [6,7,8,9,10,11].

Researchers have also found that the release of reactive oxygen species (ROS) associated with oxidative stress in response to physical activity is one the most prevalent causes of DOMS [12,13]. Eccentric exercise tends to generate especially intense muscle soreness by increasing the rate of oxidative stress, outstripping our antioxidant capacity [14,15]. One approach to this problem has centered on supplementation (SUP) with dietary antioxidants based on their known ability to stabilize these free radicals and the ROS produced by the body’s oxidative stress mechanisms [16].

While it has been long known that high concentrations of free radicals lead to oxidative damage, it has more recently been shown that the increased ROS levels induced by exercise also play a physiological role [17,18,19]. ROS and reactive nitrogen species (RNS) behave as molecular messengers through their interactions with redox-sensitive proteins that regulate processes such as insulin sensitivity, growth factor signaling, vasodilatation, and the immune response [18]. Moreover, there is mounting evidence to suggest that both radical species also play a role in adaptations to exercise through their actions as signals that modulate mitochondrial biogenesis, antioxidant defense, and hypertrophy [19].

Vitamin C (VitC) has antioxidant properties, as it interacts directly with free radicals and is able to reduce lipid peroxidation, which causes cell membrane damage [20]. Vitamin E (VitE) is also able to minimize lipid peroxidation and prevents the propagation of newer cycles of lipid radicals [21]. Both these vitamins act as direct antioxidants by regulating redox levels through the scavenging of ROS [22]. Several meta-analyses and systematic reviews have analyzed the effects on DOMS of other vitamins besides VitC and VitE, as well as their combination with other antioxidants and their effects [23,24]. However, these reviews have found mixed evidence that SUP with some antioxidants could be a solution to the problem of muscle soreness (MS). This review sought to identify studies that have included SUP with VitC and/or VitE in combination with an eccentric exercise protocol to assess the possibility that this intervention could help diminish DOMS and MS in healthy individuals. The purpose of our systematic review was to analyze the possible effects of chronic and/or acute SUP with VitC and/or VitE on DOMS and MS produced as a side effect of exercise in healthy populations of athletes, as well as of physically active and inactive subjects.

## 2. Materials and Methods

This systematic review assesses the effects of SUP with VitC and/or VitE on DOMS and MS produced as a side effect of exercise in healthy populations. The study protocol was registered at the Joanna Briggs Institute. The review was written following Preferred Reporting Items for Systematic Reviews and Meta-Analyses (PRISMA) guidelines.

### 2.1. Eligibility Criteria

Studies were identified according to PICOS model eligibility criteria, which consider the factors of population (P), intervention (I), comparators (C), and outcomes (O), along with study design (S) [25]. Participants of selected studies had to be both males and females aged between 16 and 80 years. All populations had to be healthy with no past history of injuries and no previous or recent intake of vitamin supplements. Participants of the different studies varied in that they were either sedentary, moderately active, or athletes. The interventions of the studies included chronic and/or acute SUP with VitC and/or VitE combined with a training protocol to assess the reactions of the muscles after these exercises. Comparators were the differences observed in the placebo (PLA) versus SUP groups. Though some studies measured a variety of outcomes, their primary focus had to be on muscle soreness and/or DOMS. Some studies examined different outcomes such as markers of muscle damage and oxidative stress, but these had to also include MS and/or DOMS. All study designs were required to fall in the category of primary studies including randomized controlled trials, cross-over, cohort, case-control, case reports, and case series. The following studies were excluded: secondary design studies such as meta-analyses, systematic reviews, or narrative reviews; animal studies; studies conducted in injured or ill participants; studies in which VitC and/or VitE were administered with other supplements; and articles with no full-text available, opinion pieces, commentaries, and editorials.

### 2.2. Literature Search

PRISMA guidelines were used to identify the studies to be included in this systematic review [26]. The databases that were searched were PubMed, Web of Science, Medline, and Embase. The keywords used were (“DOMS” OR “delayed onset muscle soreness” OR “muscle soreness”) AND (“antioxidant” OR “vitamin c” OR “vitamin e” OR “ascorbic acid” OR “alpha tocopherol”) AND (supplementation). All original full-text articles in English or translated to English published from January 2000 to June 2020 were considered.

### 2.3. Search Strategy

Initially, all articles identified through the database search or manual search were assessed for duplicates, and these were removed. Then, in a two-step screening process, all titles and abstracts were first read to identify relevant articles according to the search and eligibility criteria. In the second step, full-text articles were reviewed to determine if they met the optimal inclusion criteria. Fourteen studies were finally included.

### 2.4. Data Extraction

From the identified studies, specific information was extracted including: authors and year of publication; participant characteristics (level of activity or sports discipline, number of subjects, age, gender, and health status); intervention protocol (vitamin, dose, and period of SUP, type of sport/exercise); and main outcomes: DOMS, MS, perceived muscle soreness (PMS), skeletal muscle damage, and oxidative stress biomarkers, including creatine kinase (CK), myoglobin, total glutathione (TGSH), oxidized glutathione (GSSG), malondialdehyde (MDA), and thiobarbituric acid (TBA); plasma levels of VitC and VitE; and exercise performance.

### 2.5. Quality Assessment and Risk of Bias

Cochrane Collaboration Guidelines were used to assess the methodological quality of the selected studies [27]. The Cochrane Risk of Bias tool for randomized clinical studies is based on seven domains: sequence generation and allocation concealment (selection bias), the blinding of participants and personnel (performance bias), the blinding of outcome assessment (detection bias), incomplete outcome data (attrition bias), selective reporting (reporting bias), and other sources of bias (other bias). Risk of bias was categorized as low, high, or unclear.

## 3. Results

### 3.1. Study Selection

**Figure 1:**
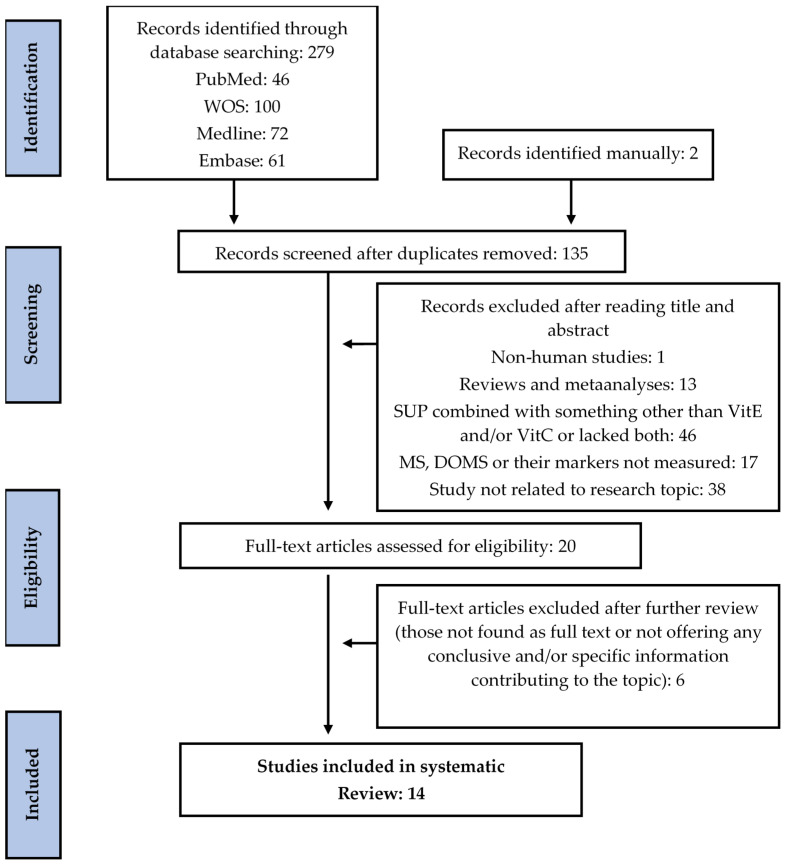
Flow diagram of literature search according to the Preferred Reporting Items for Systematic Reviews and Meta-Analyses (PRISMA) statement. SUP: supplementation; MS: muscle soreness; DOMS: delayed-onset muscle soreness; VitC: vitamin C; VitE: vitamin E; WOS: Web of Science.

Through different databases, 218 studies were initially identified, and 63 further studies were identified in the manual search. Of these records, 146 duplicates were removed and 115 studies were excluded after screening their titles and abstracts, as they did not meet the eligibility criteria. Of the 20 records remaining, two were removed as their full texts were unavailable, and four were removed according to the inclusion/exclusion criteria after examining their full texts. This left 14 randomized trials, including 10 double-blind, one cross-over, and three single-blind designs. See Figure 1 for more details.

### 3.2. Characteristics of the Studies

In the 14 reviewed studies, the total number of participants was 280, of whom 230 were male and 50 were female. In most studies, there were fewer than 25 participants; in only two were the effects of antioxidant vitamins examined in a larger group of subjects (*n* = 37 and *n* = 30) [28,29]. Across all 14 studies, the health status of the participants was good and levels of physical activity varied. The authors of five studies worked with untrained individuals [28,30,31,32,33], while participants of seven studies were described as moderately trained or physically active [29,34,35,36,37,38,39]. The remaining two studies included athletes of specific sports such as football (*n* = 1) and basketball (*n* = 1) [40,41]. Most study participants were aged between 18 and 30 years. However, in the two studies conducted in athletes, participants were under 18 years old [40,41]. The SUP protocol in the studies was classified as acute (*n* = 2) or chronic (*n* = 12). In the two studies examining the impacts of acute SUP [36,41], the antioxidant vitamin was given two-to-three hours before the eccentric exercise test. In the 12 studies in which SUP was chronic [28,29,30,31,32,33,34,35,37,38,39,40], daily vitamin supplements were taken from one month before exercise to two weeks post-exercise. The tested SUP was VitC in eight studies [28,30,31,34,36,37,38,41], it was VitE in two [32,33], and it was both vitamins in four [29,35,39,40]. The employed doses depended on the vitamin. For VitC, doses ranged from 100 to 3000 mg. For VitE, they ranged from 378 to 1400 mg. Sources of VitE were dl-α-tocopherol acetate, α-tocopherol, and alpha–beta–delta–gamma tocopherol/tocotrienol mixes.

### 3.3. Quality Assessment and Risk of Bias

**Figure 2:**
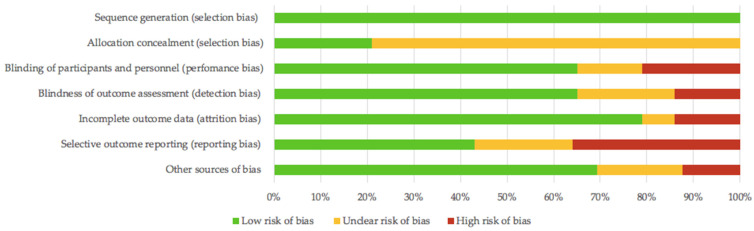
Risk of bias summary.

All 14 studies were categorized as low risk of bias in the “random sequence generation” category. In the “allocation concealment category,” 11 studies were considered of unclear risk, and the remaining three studies were considered as low risk. In the “performance and detection of bias” category, nine studies (double-blind) were defined as low risk, two studies (not defined) fell into the unclear risk category, and three studies (single-blind) were considered as high risk. In the category “incomplete outcome data,” 11 studies were considered low risk, only one study was considered to have unclear risk, and two studies considered to have a high risk of bias. In “selective outcome reporting,” six studies were defined as low risk, three studies were defined as unclear risk, and five were defined as high risk. Lastly, nine studies were described to have an overall low risk of bias, three were described to have an unclear risk, and only two were described to have a high risk. This information is detailed in Table 1 and Figure 2.

### 3.4. Results of Individual Studies

Of the 14 studies selected for this review, one study investigated the effects of acute SUP in a single exercise session [41], and another study also looked at the impacts of acute SUP but in more than one exercise session in a short period [36]. The remaining 12 studies examined the effect of chronic SUP in an acute exercise test, a single session (*n* = 10) [28,29,30,31,32,33,34,35,38,41], or a few exercise sessions per week or month (*n* = 2) [37,39] (Table 2).

#### 3.4.1. Acute Supplementation with Antioxidant Vitamins

The participants of the study by Nie and Lin [41] were 16 young, healthy male junior basketball players who were given an 800 mg dose of VitC three hours before and 21 h after performing 10 sets of full-squat jumps and 30 sets of half-squat jumps. The authors measured MS using a 10-cm visual analog scale and found that PMS in the leg extensors of both the SUP and PLA groups was not improved. In contrast, Thompson et al. [36] examined the effects of an acute dose of 1000 mg of VitC 9 in young, healthy physically active males taken two hours before LIST (Loughborough Intermittent Shuttle Test) in two sessions 14 days apart. MS was assessed using a 10-point whole-body soreness scale. Body soreness was similar in the SUP and PLA groups, so no effects of acute SUP with VitC were detected.

#### 3.4.2. Chronic Supplementation with Antioxidant Vitamins

##### Single Exercise Session

In the study by Bryer et al. [30], 18 young, healthy, untrained males took a dose of 3000 mg of VitC per day for two weeks before and four days after performing 70 eccentric elbow extensions with their non-dominant arm. The authors measured MS by rating it with a linear analog scale from 1 to 10. They found that MS increased in both the SUP and PLA groups, and there was a significant reduction in MS score in the first 24 h in the SUP group. Overall, MS was significantly higher in the PLA group across all time points.

The participants of the study by Thompson et al. [38] were 14 young, healthy, physically active men who were given 200 mg of VitC per day immediately before and three days after 30 min of downhill running on a motorized treadmill. These researchers measured MS using a 0–100 mm scale that had no adjectives or divisions except for normal on the left and very sore on the right. Results indicated no significant difference in MS rating between the SUP and PLA groups at any time point after exercise. Likewise, Close et al. [34] examined 20 young, healthy physically active men who took 1000 mg of VitC per day two hours before and 14 days after 30 min of downhill running on a motorized treadmill. DOMS was assessed using a visual analog scale and pressure algometry. Ultimately, similar mean ratings of soreness were recorded at all time points in their SUP and PLA groups.

In a population of 24 young, healthy untrained men and women, Conolly et al. [31] assessed the effects of a 1000 mg dose of VitC taken three times per day three days before and five days after 40 maximal eccentric elbow flexor contractions. DOMS was induced and measured as strength, flexibly, pain, and point tenderness assessments. The authors concluded that a VitC SUP protocol for eight days was ineffective in protecting against selected markers of DOMS. Similarly, Rahmani et al. [28] established two different SUP and two different PLA groups in 37 young, healthy untrained women. Doses of 100 or 200 mg of VitC per day were administered to the SUP groups chronically and immediately before and 24 and 48 h after performing 70 eccentric contractions of the triceps muscle with their non-dominant side on a modified arm curl machine. PMS was measured as soreness graded on a 30-point scale. According to their results, the ingestion of both doses of VitC for three days was ineffective in reducing DOMS markers post-exercise.

Two other studies used only VitE as the SUP for their protocol. Silva et al. [33] conducted their study in 21 young, healthy untrained men. The subjects were given 800 IU of VitE per day for 14 days before and seven days after completing three sets of flexion and extension of the elbow on the Scott bench at two-minute intervals until exhaustion. MS among participants was assessed using a 10-cm visual analog scale. The results revealed a significant increase in MS in both the SUP and PLA groups on days two, four, and seven after eccentric contractions. Scores in the SUP group were lower than in the PLA group four and seven days after eccentric exercise. Similarly, Kashef [32] measured DOMS in 20 young, healthy sedentary men who took 400 IU of VitE per day post-dinner time for 28 days before and two days after 10 min of bench leg step-ups with both legs at a rate of one step per second. DOMS was measured with the Likert scale of muscle soreness to obtain mean scores for the participants. Across all time points, VitE SUP was ineffective in ameliorating markers of DOMS induced by eccentric exercise.

In three studies, the combined effects of VitC and VitE were explored. De Oliveira et al. [40] examined 21 young, healthy male football players. The dose given was 500 mg of VitC and 400 IU of VitE per day for seven days before and seven days after performing plyometric jumps with strength resistance sets until exhaustion. DOMS was determined using a visual analog scale for soreness and the rating was between 1 and 10. After reviewing results, it was concluded that the antioxidants did not significantly reduce DOMS during the recovery week. Likewise, Shafat et al. [35] examined 12 young, healthy moderately active males given 500 mg of VitC and 1200 IU of VitE per day for 30 days before and seven days after a test consisting of 30 eccentric contractions of knee extensions with the dominant leg. DOMS was graded from 8 to 80 using visual analog questionnaire scales for a total of eight body sites. The researchers concluded that VitE plus VitC SUP did not modify the time course of muscle soreness. Another study testing the use of both vitamins was conducted by Bloomer et al. [29] in 30 young, healthy, trained men. Participants were administered 1000 mg of VitC and 378 mg of VitE per day for 14 days before and two days after executing 10 sets of 10 repetitions of the barbell Smith machine bench press exercise. MS was measured with a 10-point cm visual analog scale, and subjects reported their perceived muscle soreness. Ratings showed there were no significant differences in the time course of MS between the SUP and PLA groups. Thus, the intake of both antioxidants seems ineffective in reducing MS post-exercise.

##### Chronic Exercise Protocol

In two of the reviewed studies, a training intervention including more than one session of exercise per week or month was carried out. The participants of the study by He et al. [39] were 22 young, healthy, moderately trained men. Researchers supplied the SUP group with 1000 mg of VitC and 400 IU of VitE per day right before each session and for two days after them. DOMS was determined using the Rodenburg (1993) soreness rating scale (0–6). MS scores indicated that DOMS was significantly lower in SUP than PLA 24 h post-exercise in both the quadriceps and tibialis anterior. In a similar manner, Thompson et al. [37] administered 200 mg of VitC dissolved in a 500 mL drink to 16 young, healthy, physically active males twice per day immediately after and two days after performing LIST. MS was assessed using a visual MS scale ranging from1 to 10. The mean score for five muscular sites in the leg flexors and extensors indicated no difference in MS at any time point between the SUP and PLA groups.

## 4. Discussion

The main finding of this systematic review was that SUP with antioxidant vitamins seems to have little to no effect on delayed onset muscle soreness. Out of the 14 studies reviewed here, only three were able to detect significantly improved MS across all time points when participants were given a SUP of VitC and/or VitE when compared to a PLA group [30,33,39]. Accordingly, we can state that only 21% of the SUP protocols tested in the reviewed studies returned favorable results. In contrast, 11 studies identified no difference in the markers PMS, MS, or DOMS in response to vitamin intake [28,29,31,32,34,35,36,37,38,40,41]. As an overall conclusion, the intake of VitC and/or VitE seems ineffective against DOMS/MS and for aiding recovery after exercise. Thus, these vitamins are not beneficial ergogenic aids to protect against muscle damage. In this discussion, we further analyze the different aspects of the investigations reviewed that could have influenced their results.

### 4.1. Comparison Statement

The effects of vitamin SUP may have been associated with too few comparators. Overall, the studies shared characteristics such as small sample size, age group, gender, health status, and past injuries. As participants were considered healthy and free of injuries, we can rest assured that any symptoms post-eccentric exercise did not arise from an underlying health problem.

All the investigations included fewer than 40 participants in the age range from 16.7 ± 0.2 to 28.4 ± 1.3 years, which made it a young population. According to some research, eccentric exercise contractions generate greater damage and side effects in the muscles of older subjects compared to younger individuals [42]. Thus, the age group chosen for the studies was appropriate to minimize the overestimation of the results. Male participants were predominant, and only two studies included female subjects [28,31]. A review of sex differences in exercise-induced muscle pain and muscle damage markers suggested no variation in DOMS according to sex [43]. However, it did suggest that women may experience more muscle pain after exercise-induced damage, which could explain why the studies in which antioxidant SUP was found to be effective only had male participants [30,33,39]. Levels of physical activity did nevertheless vary between the study participants. In the studies by De Oliveira et al. [40] and Nie and Lin [41], in which participants were professional basketball and football players, SUP was observed to not be of much help for their participants. According to a study in trained vs. untrained individuals in conditions of oxidative stress post eccentric exercise, trained individuals emerged as more likely to experience lower DOMS levels, which might also make them less receptive to SUP treatment for this condition [44]. According to those studies detecting no minimizing of MS, we can infer that untrained to moderately active people have a greater chance of improving their post-exercise muscular symptoms through the intake of VitC and/or VitE.

### 4.2. Supplementation Protocols

The different reviewed studies showed a great diversity of implemented methods. All study designs were randomized and controlled with at least the blinding of the participants. The durations of the interventions were under two months. There did not appear to be an optimal time-frame that seemed most efficient for SUP to act on DOMS. However, in the studies detecting a reduction in MS, SUP commenced two weeks before and finished two-to-seven days after the exercise test. The authors of one such study stated that although further experimentation is necessary, pretreatment with VitC might be able to reduce post-exercise MS [30]. This noted effect could, nevertheless, be related to the production of pain and inflammatory enzymes like prostanoids. Shafat et al. [35] and Kashef [32], on the other hand, found no reducing effect on DOMS of SUP given 28–30 days before exercise. Only two of the studies reviewed here tested the impacts of acute SUP protocols. Thus, while Nie and Lin [41] noted the reduced release of the muscle damage marker CK, both investigations were unsuccessful in reducing MS [36]. The findings of a review of the impacts of both VitE and VitC on exercise-induced muscle damage indicated that acute SUP may protect against muscle damage and improve recovery [45]. Despite this, we observed reductions in DOMS in those studies following a chronic SUP protocol before and after eccentric exercise. Accordingly, there could be a pattern whereby administering antioxidants too late before exercise might reduce the efficacy of SUP. In the studies by Thompson et al. [37] and Thompson et al. [38], SUP was given either before or immediately after the LIST test and continued for no longer than three days. This could perhaps explain why most chronic SUP regimens failed to diminish DOMS. Regarding SUP dose, there is no clear or conclusive evidence as to which regimen behaves better. Most studies examined SUP doses that exceeded the daily recommendations for each vitamin. For instance, two studies used 3000 mg of VitC per day in similar subject groups, but the study by Bryer et al. [30] was the only one to achieve a reduction in MS [31]. Similarly, He et al. [39] were able to significantly lower DOMS in their SUP group when compared to PLA at 24 h post-exercise using 1000 mg of VitC and 400 IU of VitE. Other authors also used 1000 mg of VitC [34,36], one in combination with VitE [29], but this regimen seemed to not be able to significantly improve DOMS markers. Overall, there is insufficient information to conclude whether a specific vitamin SUP protocol of a given duration will be more effective than another at improving MS, as the three studies reporting a significant beneficial effect of vitamin intake used different SUP protocols [30,33,39]. A recent meta-analysis reviewed the effect of VitC SUP on inflammation, muscle soreness, and strength following acute exercise in a healthy population. The results of seven of the studies indicated that VitC SUP had no effect on muscle soreness [23].

### 4.3. Eccentric Exercise Regimen

When addressing the exercise test protocols of the studies, there were variations in the number of sessions, the time between them, and the type of eccentric exercise used. Most studies included a single exercise session; only three implemented more than one. In two studies, physically active subjects completed the LIST in two sessions: Thompson et al. [37] separated them by three days, while in the other study by Thompson et al. [36] the tests were conducted 14 days apart. Under these circumstances, neither acute nor chronic SUP was noted to have MS-lowering effects after exercise [36,37]. Despite this, He et al. [39] had their moderately trained participants complete 40 min of downhill running on a treadmill (−10% grade) at 65–70% of the maximal oxygen consumption (VO_2max_) on two separate occasions at the same time of day separated by three weeks. This study found that VitC and VitE SUP significantly reduced DOMS 24 h after running in both the quadriceps and tibialis anterior [39].

Contrastingly, the studies with single exercise sessions could be divided into those that used upper body eccentric exercises and those that used lower body ones. Four studies focused on lower body exercises: Kashef [32] had their sedentary participants doing leg step-ups, Shafat et al. [35] had their moderately active subjects performing knee extensions, and in the last two by De Oliveira et al. [40] and Nie and Lin [41], athletes undertook full-squat jumps and plyometric jumps combined with strength resistance, respectively. All failed to demonstrate a significant reduction in DOMS in the SUP group at any time point after exercise. Two additional studies focused on lower extremities—those by Thompson et al. [38] and Close et al. [34]. These authors had physically active individuals do downhill running on a treadmill for 30 min, yet both were also unsuccessful at attenuating MS. Several similarities were noted between the studies based on upper body exercises. In the study by Bryer et al. [30] in which untrained participants completed 70 eccentric triceps extensions of the non-dominant limb, MS was improved after arm contractions. However, Rahmani et al. [28], following a similar training protocol involving 70 eccentric contractions on a modified arm curl machine, observed no effects of SUP on DOMS post-exercise. Silva et al. [33] had untrained participants complete three sets of flexion and extension of the elbow on the Scott bench at two minute intervals until exhaustion. Results revealed that MS was reduced in the SUP compared to PLA group at four and seven days after exercise. In contrast, Conolly et al. [31] and Bloomer et al. [29], using similar training protocols of the elbow flexors and bench press exercises, detected no differences between groups in minimizing DOMS. It should be nevertheless noted that while some participants were familiarized with the exercises, some were not or this was not specified. This might be important, as there are some data suggesting that a single exercise session beforehand could have a prophylactic effect on MS [46]. Considering all these training variables, there does not seem to be a clear pattern indicating whether or not a specific type of exercise will work better for assessing and reporting MS, as the reviewed studies varied widely in terms of inducing DOMS.

### 4.4. Variables besides DOMS

Most of the reviewed studies measured different dependent variables aside from DOMS to find out if SUP with antioxidants was able to lessen oxidative muscle damage, preventing impaired muscular function and enhancing recovery. The studies of Bryer et al. [30] and He et al. [39] involving VitC SUP or VitC plus VitE SUP found their participants to have lower DOMS in the experimental group, as well as lower CK levels in comparison to their PLA group. In contrast, Nie and Lin [41] observed SUP with VitC to be effective at lowering oxidative markers like CK, but they suggested that this marker is independent of MS. Further, De Oliveira et al. [40] found that antioxidant SUP could inhibit some oxidative stress, as reflected by reduced lipid peroxidation markers like MDA and GSSG but unsuccessfully reduced CK or minimized DOMS during muscle recovery. Similarly, muscle performance variables such as maximal voluntary contraction (MVA), eccentric contractile torque, vertical jump, agility, sprint time, fatigue index, and peak power (PP) were assessed after SUP in some studies [29,31,32,35,36,37,38,40]. Many of these studies showed that SUP with vitamins did not speed up recovery or prevent a decline in muscular function variables [29,31,36,38]. However, Shafat et al. [35] did observe a lower decline in isometric maximum voluntary contraction force (MVC) but no difference in MS in their participants taking antioxidant SUP post eccentric contractions. Collectively, these data suggest that VitC and/or VitE SUP have no beneficial impacts on muscular factors such as damage markers, function, performance, recovery, nor are they effective at attenuating DOMS.

### 4.5. Limitations

Despite meaningful evidence emerging from some studies of a link between antioxidant SUP and improved DOMS, some key aspects that could have influenced results were ignored in some trials. Variations in study designs may have significantly impacted their results. Most studies included a relatively small sample size. While the design of the study by Thompson et al. [38] was cross-over, this study only had nine participants. The majority of designs were double-blind, but at least three were single-blind ones, which could have biased the reporting of results [28,32,35]. Similarly, the populations of chosen participants noticeably varied in their levels of physical activity as did VitE and/or VitC SUP protocols, thus precluding a uniform pattern of investigation and generating conflicting results. In terms of quality of evidence, for most studies, this was classified as low-quality due to the mentioned study design flaws. Moreover, most studies measured MS using a visual analog scale, which is, of course, subject to individual bias.

Another possible limitation was that the dietary intake of the participants, as not all the studies measured their food intake before the experiment. Though there were no differences found in the consumption of dietary vitamins between participants, food recall questionnaires are still open to biased responses. Another factor that was not accounted for was sleep. Research has shown that poor sleep or sleep deprivation increases the sensitivity and perception of pain, which might have hindered perceptions of DOMS in participants [47]. Another limitation is that the estrogen levels of the female participants were not measured, so it is not really clear if SUP was effective at reducing DOMS in this gender group [28,31] because elevated estrogen levels may have a protective effect on muscle tissue after exercise [48]. Additionally, a genetic predisposition for DOMS was not accounted for in any of the studies. There have been reports that lower production levels of genes like alpha-actinin-3 (ACTN3) and myosin light-chain kinase (MLCK) will make a person more susceptible to muscle damage and subsequently to DOMS [49,50].

If we compare our findings to those of meta-analyses and systematic reviews that have touched upon the topic of our review, it seems that despite some significant evidence in a few studies, most of the evidence points to no DOMS-attenuating effect of antioxidant SUP with VitC and/or VitE. A meta-analysis of randomized clinical trials examining the effects of VitC on oxidative stress, inflammation, muscle soreness, and strength following acute exercise concluded that this type of SUP intervention does not lower levels of CK, CRP, cortisol, and MS, nor can it improve muscle strength [23]. Another systematic review addressing a variety of antioxidants to prevent and reduce muscle soreness after exercise found that SUP with different types of antioxidants including VitE and VitC did not lead to a clinically significant reduction in DOMS [24]. The findings of both these reviews were consistent with our results.

## 5. Conclusions

Only in 21% of the reviewed studies was SUP with antioxidant vitamins found to have a positive effect in diminishing DOMS and/or MS. The remaining 79% of the studies detected no differences in PMS, MS, or DOMS markers in their participants. Thus, independently of the intake of VitC and/or VitE, supplementation did not emerge as effective in reducing DOMS/MS and aiding recovery after eccentric exercise. We may therefore conclude that SUP with these vitamins has no ergogenic benefits for preventing muscle soreness.

We found some relatively weak evidence that acute and/or chronic SUP with VitC and/or VitE could have beneficial effects on DOMS produced 24–72 h after eccentric exercise. Because of various factors such as study design, SUP protocols, training regimen, and other limiting variables, the question remains as to whether this ergogenic approach is truly effective at diminishing MS or if outcomes are related more to preventing lipid peroxidation, as suggested by a few studies. These limitations need to be addressed in further research on this topic.

## Figures and Tables

**Table 1:** Risk of bias.

Study	Sequence Generati0n (Selection Bias)	Allocation Concealment (Selection Bias)	Blinding of Participants and Personnel (Performance Bias)	Blindness of Outcome Assessment (Detection Bias)	Incomplete Outcome Data (Attrition Bias)	Selective Outcome Reporting (Reporting Bias)	Other Sources of Bias
Bryer et al., 2006 [30]	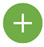	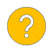	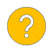	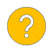	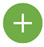	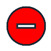	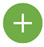
Conolly et al., 2006 [31]	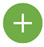	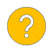	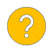	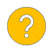	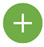	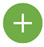	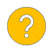
Close et al., 2006 [34]	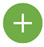	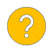	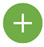	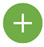	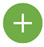	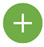	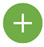
De Oliveira et al., 2019 [40]	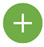	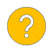	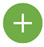	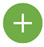	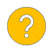	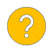	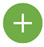
He et al., 2015 [39]	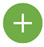	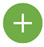	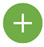	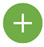	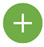	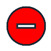	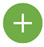
Rahmani et al., 2008 [28]	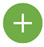	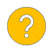	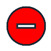	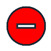	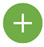	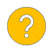	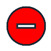
Thompson et al., 2004 [38]	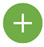	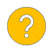	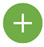	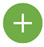	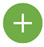	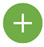	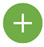
Shafat et al., 2004 [35]	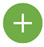	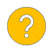	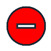	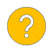	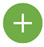	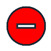	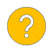
Bloomer et al., 2007 [29]	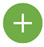	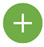	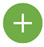	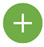	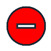	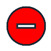	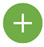
Thompson et al., 2001 [36]	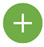	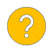	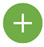	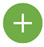	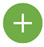	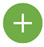	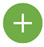
Kashef 2018 [32]	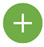	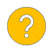	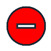	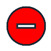	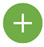	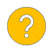	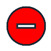
Thompson et al., 2003 [37]	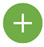	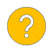	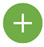	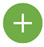	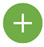	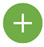	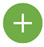
Silva et al., 2010 [33]	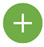	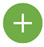	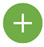	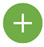	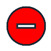	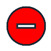	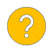
Nie and Lin 2004 [41]	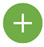	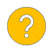	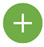	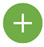	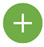	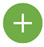	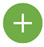

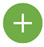
 Low risk of bias; 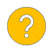 Unclear risk of bias; 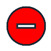 High risk of bias.

**Table 2:** Results and details of the reviewed studies.

Study	Subjects	Supplementation and Timing	Exercise	DOMS, MS, and PMS Variables	Variables other than DOMS, MS, PMS	Results
Bryer et al., 2006 [30]	18 healthy untrained males: SUP (*n* = 8) 21.4 ± 0.8 years PLA (*n* = 10) 24.4 ± 1.7 years	VitC (3000 mg) per day for 18 days 2 weeks pre-exercise and 4 days post-exercise	70 eccentric elbow extensions with non-dominant arm	MS: linear scale from 1 to 10 Before exercise, immediately after, and 4, 24, 48, 72, and 96 h after exercise.	MIF ROM CK GSSG TGSH	MS → PLA > SUP across all time points. MS → increased in both groups yet was significantly reduced for the first 24 h in the VitC SUP group.
Conolly et al., 2006 [31]	24 healthy untrained males and females: SUP (*n* = 12) 22.3 ± 3.9 years PLA (*n* = 12) 22.6 ± 4.6 years	VitC (1000 mg) 3 times per day for 8 days 3 days pre-exercise and 5 days post-exercise	40 (2 sets x 20 reps) maximal eccentric elbow flexor contractions	DOMS: strength, flexibly, pain, and point tenderness assessments Before exercise and 24, 48, 72, and 96 h after exercise and DOMS induction.	-	DOMS → VitC is ineffective in protecting against selected markers of DOMS.
Close et al., 2006 [34]	20 healthy physically active males: SUP (*n* = 10) 24.2 ± 1.5 years PLA (*n* = 10) 22.1 ± 0.4 years	VitC (1000 mg) per day for 15 days 2 h pre-exercise and 14 days post-exercise	Downhill running on motorized treadmill for 30 min	DOMS: VAS and pressure algometry Ratings of DOMS were presented as mean soreness of eleven assessed sites 2 h before exercise as well as 1, 2, 3, 4, 7, and 14 days post-exercise.	Plasma VitC ROS MDA TGSH MF	DOMS→ SUP = PLA at all time points.
De Oliveira et al., 2019 [40]	21 male football players: SUP (*n* = 11) 16.7 ± 0.3 years PLA (*n* = 10) 17.0 ± 0.3 years	VitC (500 mg) and VitE (400 IU of α-tocopherol) per day for 15 days 7 days before and 7 days after exercise	Plyometric jump and strength resistance set to exhaustion	DOMS: VAS before and 24, 48, and 72 h post-exercise.	Plasma VitC Plasma VitE MDA CK GSSG TGSH	DOMS → SUP = PLA during recovery week.
He et al., 2015 [39]	22 moderately trained males: SUP (*n* = 11) 20.5 ± 2.3 years PLA (*n* = 11) 21.3 ± 4.0 years	VitC (1000 mg) and VitE (400 IU) per day for 14 days Before each session and 2 days post session	40-min downhill running on treadmill performed at the same time of day 2 different sessions separated by 3 weeks	DOMS: Rodenburg (1993) rating of soreness scale. Rated immediately after, and 24, 48, and 72 h after each trial.	CK ORAC	DOMS → PLA > SUP at 24 h after exercise in both the quadriceps and tibialis anterior.
Rahmani et al., 2008 [28]	37 healthy non-athletic females (22.02 ± 1.54 years) 4 groups: SUP 100 mg (*n* = 9) SUP 200 mg (*n* = 10) PLA (*n* = 9) Control (no SUP or PLA) (*n* = 9)	VitC (100 mg) and VitC (200 mg) per day for 3 days Immediately before exercise and 24 and 48 h post-exercise	70 eccentric contractions of the triceps muscle of the non-dominant side on a modified arm curl machine	PMS: soreness graded using a 30-point scale Evaluated 4 times on Day 1: 1 h pre exercise and 1h post-exercise Day 2: 2 h after SUP Day 3: 2 h after SUP.	CK ROM MEC EROM	DOMS → SUP (100 and 200 mg) = PLA.
Thompson et al., 2004 [38]	14 physically active males: SUP (*n* = 7) 25.3 ± 1.4 years PLA (*n* = 7) 22.6 ± 1.7 years	VitC (200 mg) twice a day for 14 days Before exercise and 3 days post-exercise	Downhill running on motorized treadmill for 30 min	MS: soreness scale 0–100 mm Before exercise and 24, 48, and 72 h post-exercise.	Plasma VitC CK Myoglobin Interleukin-6 MF	MS → SUP = PLA at all time points after exercise.
Shafat et al., 2004 [35]	12 healthy moderately active males: SUP (*n* = 6) 25.0 ± 7.5 years PLA(*n* = 6) 20.6 ± 1.1 years	VitC (500 mg) and VitE (1200 IU of α-tocopherol) per day for 37 days 30 days pre-exercise and 7 days post-exercise	30 eccentric contractions of knee extensions with dominant leg	DOMS: VAS for a total of 8 body sites Before exercise, immediately after and every day for 7 days post-exercise.	MVC MF	MS→ SUP = PLA at all time points after exercise and recovery.
Bloomer et al., 2007 [29]	30 healthy trained males 4 groups: No prior exercise SUP (*n* = 7) 23 ± 2 years No prior exercise PLA (*n* = 8) 25 ± 5 years Prior exercise SUP (*n* = 8) 22 ± 2 years Prior exercise PLA (*n* = 7) 25 ± 4 years	VitC (1000 mg) and VitE (378 mg mixed tocopherols/tocotrienols) two capsules per day for 16 days 14 days pre-exercise and 2 days post-exercise	10 sets of 10 repetitions of the barbell Smith machine bench press exercise	MS: VAS Before exercise, immediately after and 24 and 48 h post-exercise.	MIF MF CK CRP Protein carbonyls Peroxides	MS→ SUP = PLA at all time points after exercise.
Thompson et al., 2001 [36]	9 healthy physically active males 28.4 ± 1.3 years	VitC (1000 mg) per day for 1 day 2 h pre-exercise	LIST two sessions separated by 14 days	MS: VAS for whole-body Before exercise and 24, 48, and 72 h post-exercise.	Plasma VitC MDA CK Uric Acid Cortisol Total Iron	MS→ SUP = PLA at all time points after exercise.
Kashef 2018 [32]	20 healthy sedentary males: SUP (*n* = 10) 22.4 ± 2.5 years PLA (*n* = 10) 22.7 ± 2.2 years	VitE (400 IU) per day for 30 days Post-dinner time 28 days pre-exercise and 2 days post-exercise	10 min of bench leg step ups with both legs at a rate of 1 step per second	DOMS: Likert scale of muscle soreness MS measured as mean score Immediately after and 48 h post-exercise.	PP CRP CK	DOMS→ SUP = PLA at all time points after exercise.
Thompson et al., 2003 [37]	16 healthy physically active males: SUP (*n* = 8) 23.6 ± 1.4 years PLA (*n* = 8) 24.3 ± 1.7 years	VitC (200 mg) dissolved in a 500 mL drink twice a day for 3 days Immediately after and 2 days post-exercise	LIST 2 sessions separated by 3 days	MS: VAS Mean score for five sites Before exercise and 24, 48, and 72 h post-exercise.	Plasma VitC MDA CK Serum cortisol Interleukin-6 Uric acid	MS→ SUP = PLA at time points in leg flexors and extensors.
Silva et al., 2010 [33]	21 healthy untrained males: SUP (*n* = 11) 22.5 ± 4 years PLA (*n* = 10) 22.5 ± 4 years	VitE (800 IU of D-α-tocopherol acetate) per day for 21 days 14 days pre-exercise and 7 days post-exercise	3 sets of flexion and extension of the elbow on the Scott bench at 2 min intervals until exhaustion	MS: VAS Before exercise and 2, 4, and 7 d post-exercise.	LDH Lipid peroxidation Protein carbonylation Interleukins	MS→ PLA > SUP at 4 and 7 days after eccentric exercise.
Nie and Lin 2004 [41]	16 healthy male junior basketball players: SUP (*n* = 8) 16.7 ± 0.3 years PLA (*n* = 8) 16.5 ± 0.2 years	VitC (800 mg) per day for 1 days 3 h pre-exercise and 21 h post-exercise	Eccentric exercise trial (10 sets of full-squat jumps at maximum exertion and 30 sets of half-squat jumps)	PMS: VAS Before exercise, immediately after and 24 and 48 h post-exercise.	Plasma VitC MDA CK	MS → SUP = PLA at all time points after exercise.

C-reactive protein (CRP), creatine kinase (CK), delayed onset muscle soreness (DOMS), elbow range of motion (EROM), international units (IU), isometric maximum voluntary contraction force (MVC), lactate dehydrogenase (LDH), Loughborough Intermittent Shuttle Test (LIST), maximum isometric force (MIF), maximal eccentric contraction (MEC), muscle function (MF), muscle soreness (MS), oxidized glutathione (GSSG), oxygen radical absorbance capacity (ORAC), peak power (PP), perceived muscle soreness (PMS), placebo group (PLA), plasma malondialdehyde (MDA), range of motion (ROM), reactive oxygen species (ROS) supplemented group (SUP), total glutathione (TGSH), visual analogic scale (VAS), vitamin C (VitC), and vitamin E (VitE).
